# Assessing the prevalence of generalized anxiety disorder in a multicultural medical education setting in Saudi Arabia during the COVID-19 pandemic

**DOI:** 10.3389/fpsyt.2024.1359348

**Published:** 2024-07-19

**Authors:** Nader Ashraf, Tarek Ziad Arabi, Alhomam Dabaliz, Bader Abou Shaar, Omar Javed Baqal, Rand Mohammad Taha, Eman Javed Baqal, Mohamad Salim Alkodaymi, Akef Obeidat, Abderrahman Ouban

**Affiliations:** ^1^ College of Medicine, Alfaisal University, Riyadh, Saudi Arabia; ^2^ Department of Radiology and Biomedical Imaging, Yale School of Medicine, New Haven, CT, United States; ^3^ Department of Internal Medicine, Mayo Clinic Arizona, Pheonix, AZ, United States

**Keywords:** generalized anxiety disorder, mental health, medical student, Middle East, medical education

## Abstract

**Introduction:**

Mental health among medical students is an area that has been increasingly garnering world-wide attention. Yet, despite this increased attention, research related to anxiety disorders in Saudi Arabia remain limited. This study aims to address this gap by assessing the prevalence of generalized anxiety disorder (GAD) as well as explore its association with students’ sociodemographic factors and academic performance among medical students in a Saudi Arabian institute that has a diverse, multicultural student body.

**Methods:**

The study takes place in a unique educational environment: a privately funded institute in Riyadh, the capital of Saudi Arabia, with 32% of its student body comprising international students from over 40 countries, during the COVID-19 pandemic. The study deploys the use of a survey that was sent to the students at this institute via their institutional emails. A survey containing 33 items assessing demographics, GAD using GAD-7 questionnaire, stress-relief measures, online learning experience during the pandemic, mental disorders, anxiety associated with the pandemic, two open-ended questions, and one item assessing sleep difficulty.

**Results:**

The results of the survey showed that a significant majority of the responders’ scores in the GAD-7 assessment were in the range of moderate to severe anxiety. The results also suggest that low cGPA and the first academic years are significantly associated with higher GAD-7 scores. Students found severe time constraints, difficult educational content, and lack of psychological support to be major sources of stress. Furthermore, students suggested providing an in-house psychologist, personalized feedback, and guidance on study tactics as methods to reduce anxiety.

**Conclusion:**

These findings, among others, suggest a need for further studies and research in this field to uncover broader patterns and inform targeted mental health support strategies.

## 1 Introduction

Mental health among medical students is a critical area of concern, with studies indicating a high prevalence of mental health issues in this population due to the demanding nature of medical education (1). These challenges, including academic pressures, social isolation, and financial stress, contribute significantly to mental health disorders (2). While much of the focus in literature has been on depression, anxiety disorders, and specifically Generalized Anxiety Disorder (GAD), are increasingly being recognized as significant concerns (3).

GAD, characterized by persistent, excessive, and unrealistic worry (4) emerges in medical students due to various factors such as personality traits (5), academic workload (6), sleep deprivation (7), and financial pressures (7). The impact of patient deaths (8) and student mistreatment (9) are also notable. The COVID-19 pandemic has exacerbated mental health issues among medical students, highlighting the need for robust mental health support systems (10, 11). Anxiety can significantly impact academic performance and professional competence, with cognitive functions, particularly in learning environments, being adversely affected (12, 13).

Despite the increasing recognition of GAD among medical students, research in the Middle Eastern context, particularly in Saudi Arabia, remains limited (14, 15). GAD is estimated to be prevalent in approximately one-third of medical students in Saudi Arabia (14). Furthermore, this study is set against the backdrop of a region where mental health often carries a significant stigma (16–18). In Saudi Arabia, like much of the Middle East, there is a marked reluctance to discuss mental health issues openly, often viewed as a taboo subject (19, 20). This societal stigma can lead to underreporting of mental health issues and a hesitancy to seek help (19, 21). Given the diverse student body, the multicultural aspect of our institution offers an unparalleled opportunity to examine GAD in a setting that merges varied social, cultural, and educational backgrounds, potentially affecting the manifestation and perception of GAD.

This study aims to address this gap by assessing the prevalence of GAD among medical students in a Saudi Arabian institute. By exploring GAD within this unique socio-cultural milieu, this study seeks to shed light on the complexities surrounding mental health in a setting where such discussions are often limited. In doing so, we hope to contribute to the destigmatization of mental health issues in this region. The insights gained could be pivotal in developing culturally sensitive approaches to mental health care in Saudi Arabia and similar contexts.

## 2 Methodology

### 2.1 Study setting

The study takes place in a unique educational environment: a privately funded institute in Riyadh, the capital of Saudi Arabia, with 32% of its student body comprising international students from over 40 countries. This diverse cultural mix provides a distinctive setting to explore the prevalence and nuances of GAD among medical students. The multicultural aspect of our institution offers an unparalleled opportunity to examine GAD in a setting that merges varied social, cultural, and educational backgrounds, potentially affecting the manifestation and perception of GAD.

### 2.2 Survey development

To the best of our knowledge, there was no validated questionnaire dedicated to exploring generalized anxiety disorder, especially during the COVID-19 pandemic quarantine, in Saudi Arabia. Our survey encompassed 33 items: 11 items assessing stress-relief measures, eight items from the GAD 7-item (GAD-7) scale (22), six items assessing demographics, three items exploring the online learning experience during the pandemic, two items generally assessing mental disorders, two items evaluating anxiety associated with the pandemic, two open-ended questions, and one item assessing sleep difficulty. The key topics covered in the questionnaire included the following: anxiety level changes during the quarantine, difficulty sleeping due to the pandemic, memory retention and concentration changes due to online learning, and different techniques employed for anxiety relief.

Among the questions assessing anxiety changes, sleeping difficulties, and online learning during the pandemic, four were close-ended questions and two were five-point Likert scale questions. In GAD-7 scale, eight items were appraised using the 4-point Likert scale. The first seven items had the following options: not at all [0], several days [1], over half the days [2], nearly every day [3]; total score (adding all numbers) provides a possible score from 0–21. GAD-7 score range of 0–4, 5–9, 10–14, and 15–21 indicate minimal, mild, moderate, and severe anxiety, respectively. The eighth item assessed the extent of the effect that the problems mentioned in the first 7 items had on the person’s daily life. It had the following options: Not Difficult at all, Somewhat Difficult, Very Difficult, Extremely Difficult. A diagnosis of GAD should not be based on the GAD-7 scores alone; however, a score of 10 or higher indicates that further evaluation is needed.

To ensure instrument validity, an extensive literature review was performed to develop the survey items. Additionally, these items underwent evaluation and validation by a panel of subject matter experts. A preliminary study involving 30 medical students from our institution was conducted to assess the tool’s clarity and face validity, as well as to identify any technical challenges. These participants were subsequently asked to refrain from completing the survey in its final iteration to prevent repeat responses. The pilot phase indicated that the average completion time for the survey was approximately 7 minutes. Based on the feedback obtained, several survey questions were refined to enhance clarity and reduce potential confusion. We also addressed several technical issues identified during the pilot phase. The final survey was then created and disseminated using Google® Forms.

### 2.3 Study population

The study population consisted of medical students from all academic years studying in the college of medicine at our institute. Faculty, staff, graduates, and non-medical students were excluded from the study population.

### 2.4 Survey distribution

This study utilized a convenience sampling technique. A message bearing the questionnaire link was circulated via the university’s institutional email system to the target population. The survey contained an introductory paragraph that details the study’s aim and affirmed participant anonymity as well as the liberty to withdraw or decline a response entirely. We collected responses using the survey starting from August 2020 until January 2021. In March 2020, the Saudi Ministry of Education announced the closure of all schools and universities due to the pandemic, and all lectures were given using an online format. During the start of the next academic year in August 2020 and our study period, the university began implementing a hybrid teaching format, where students would take turns in small groups to attend lectures on campus, while the remainder attended the sessions online.

### 2.5 Statistical analysis

The accumulated data were organized and tabulated using Microsoft® Excel (Microsoft Corporation, Redmond, WA). Categorical data were described as counts. For quantitative data, the range, mean, and standard deviation were calculated. Statistical analysis was performed using GraphPad® Prism 9 version 9.4.1 (GraphPad Software, San Diego, CA). Shapiro-Wilk tests were used to determine if the data follow a Gaussian distribution. Due to the abnormal distribution of the data, comparisons between two or more groups were conducted using the Mann-Whitney, Kruskal-Willis, and chi-square tests. Statistical significance was set at *P* < 0.05.

### 2.6 Ethics approval

In compliance with the provisions of the Saudi Law of Ethics of Research on Living Creatures and regulations, and under the guidelines of the National Committee of Bioethics, ethics approval has been acquired from the institutional review board (Reference IRB-18098). Informed consent was obtained from all students.

## 3 Results

The calculated sample size to achieve a 95% confidence interval with a margin of error at 5% was determined to be 293, as per the methodology outlined by Pourhoseingholi et al. (23). Our survey received 431 responses comprising 37.1% (160/431) Saudi and 62.9% (271/431) non-Saudi students. The cohort included 33.4% (144/431) male and 66.6% (287/431) female students, aged between 17–28 years. First-year students were the most responsive group, comprising 34.1% (147/431) of the total responses. A majority, 66.6% (287/431), reported a cumulative GPA in the range of 3.0 to 4.0 out of 4.0. **Table 1** delineates the demographic and academic characteristics of the respondents.

**Table 1:** Baseline characteristics of survey respondents (*N* = 431).

Variable	Total, *n* (%)
**Age (Mean ± SD)**	20.68 ± 2.103
Gender	
Male	144 (33.4)
Female	287 (66.6)
Ethnic background	
Saudi	160 (37.1)
Non-Saudi	271 (62.9)
Current academic year	
1	147 (34.1)
2	80 (18.6)
3	70 (16.2)
4	68 (15.8)
5	30 (6.9)
6	36 (8.4)
Cumulative GPA	
No GPA Yet	88 (20.4)
< 3.00	56 (13)
3.00–3.24	42 (9.7)
3.25–3.49	73 (16.9)
3.50–3.74	69 (16.0)
3.75–4.00	103 (24)

Starting with mental health specifics (**Table 2**), 14.6% (63/431) of respondents acknowledged a previous diagnosis of a mental disorder, while 39.9% (172/431) suspected they might have an undiagnosed mental health condition. In terms of specific conditions, among those diagnosed, 63.5% (40/63) had anxiety disorders and 44.4% (28/63) had depression. Within the group suspecting undiagnosed conditions, anxiety disorders and depression were suspected by 52.9% (91/172) and 37.8% (65/172), respectively.

**Table 2:** Mental disorders diagnosed or self-suspected among the students (*N* = 431).

Diagnosed mental disorders		Suspicion of an undiagnosed mental disorder	
Anxiety disorders	40	Anxiety disorders	91
Depression (including major depression)	28	Depression (including major depression)	65
Attention-deficit/hyperactivity disorder	4	Attention-deficit/hyperactivity disorder	8
Obsessive compulsive disorder	3	Post-traumatic stress disorder	7
Eating disorders	2	Obsessive compulsive disorder	4
Bipolar disorder	2	Sleep disorders	4
Post-traumatic stress disorder	2	Mood disorder/cyclothymia	4
Panic disorder	2	Schizophrenia	4
Adjustment disorder	1	Bipolar disorder	3
Avoidant personality disorder	1	Eating disorders	3
Complicated grief	1	Hair pulling disorder	2
Conversion disorder	1	Panic disorder	2
Borderline personality disorder	1	Stress-related disorder	1
N/A – did not specify disorder	3	N/A – did not specify disorder	36
Total	n = 63*	Total	n = 172*

*Some students were diagnosed or suspected having more than one disorder. This number represents the number of students and not disorders.

Saudi students represented a substantial majority among both individuals diagnosed and suspected having a mental disorder (*P* < 0.01). First-year students notably reported suspecting undiagnosed mental health conditions more frequently (*P* < 0.01). Furthermore, students with lower cGPAs were significantly more likely to be diagnosed or suspect a mental health condition (*P* < 0.01). Living alone was associated with a higher likelihood of a diagnosed mental health condition, compared to living with spouse/family or roommate/s (*P* < 0.05). These associations are further detailed in **Tables 3** and **4**.

**Table 3:** Significant results of Chi-square test for factors associated with being diagnosed with a mental disorder (*N* = 431).

Variable		Diagnosed with a mental disorder	Not diagnosed with a mental disorder	P-value
**Ethnicity**	Saudi (*n* = 160)	42 (26.3)	118 (73.7)	<0.01
	Non-Saudi (*n* = 271)	21 (7.7)	250 (92.3)	
**cGPA**	No cGPA (*n* =88)	13 (14.8)	75 (85.2)	<0.01
	< 3.00 (*n* = 56)	20 (35.7)	36 (64.3)	
	3.00–3.24 (*n* = 42)	8 (19)	34 (81)	
	3.25–3.49 (*n* = 73)	11 (15.1)	62 (84.9)	
	3.50–3.74 (*n* = 69)	4 (5.8)	65 (94.2)	
	3.75–4.00 (*n* = 103)	7 (6.8)	96 (93.2)	
**Living conditions**	Alone (*n* = 53)	14 (26.4)	39 (73.6)	<0.05
	With spouse/family (*n* = 352)	47 (13.4)	305 (86.6)	
	With roommate/s (*n* = 26)	2 (7.7)	24 (92.3)	

Data reported as n (%).

**Table 4:** Significant results of Chi-square test for factors associated with being suspicious of having an undiagnosed mental disorder (*N* = 431).

Variable		Suspect having an undiagnosed mental disorder	Do not suspect having an undiagnosed mental disorder	P-value
**Ethnicity**	Saudi (*n* = 160)	86 (53.8)	74 (46.2)	<0.01
	Non-Saudi (*n* = 271)	85 (31.4)	186 (68.3)	
**Current academic year**	1 (*n* = 147)	74 (50.3)	73 (49.7)	<0.01
	2 (*n* = 80)	31 (38.8)	49 (61.2)	
	3 (*n* = 70)	27 (38.6)	43 (61.4)	
	4 (*n* = 68)	25 (36.8)	43 (63.2)	
	5 (*n* = 30)	7 (23.3)	23 (76.7)	
	6 (*n* = 36)	7 (19.4)	29 (80.6)	
**cGPA**	No cGPA (*n* =88)	49 (55.7)	39 (44.3)	<0.01
	< 3.00 (*n* = 56)	31 (55.4)	25 (44.6)	
	3.00–3.24 (*n* = 42)	16 (38.1)	26 (61.9)	
	3.25–3.49 (*n* = 73)	24 (32.9)	49 (67.1)	
	3.50–3.74 (*n* = 69)	29 (42)	40 (58)	
	3.75–4.00 (*n* = 103)	23 (22.3)	80 (77.7)	

Data reported as n (%).

**Figure 1:**
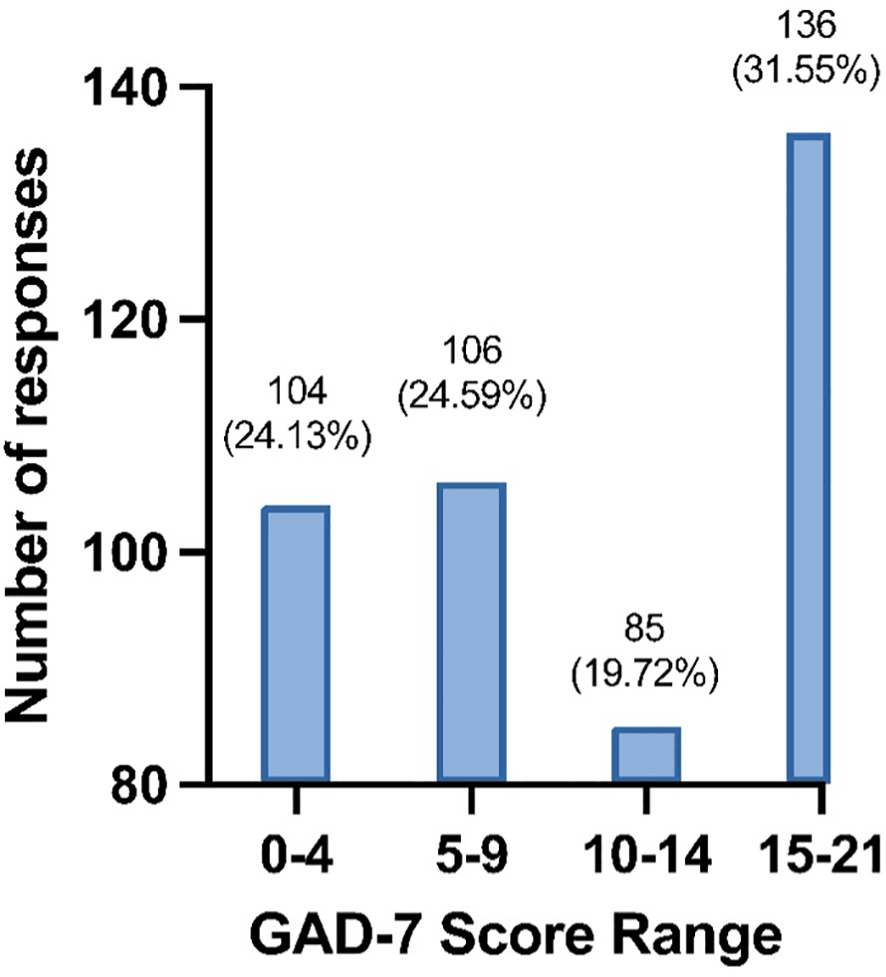
Distribution of anxiety levels among all students (*N* = 431).

The distribution of GAD-7 scores shows that the majority of students experience moderate to severe anxiety levels, with 31.6% (136/431) in the severe range (14–20) and 19.7% (85/431) in the moderate range (10–14) (**Figure 1**). Several factors were found to be associated with GAD-7 scores among medical students (**Table 5**). Being a female (11.7 ± 6.4 vs. 7.8 ± 6.4, P < 0.01), Saudi (12.1 ± 6.6 vs. 9.4 ± 6.5, P < 0.01), or a first-year student (13.1 ± 6.3, P < 0.01) correlated significantly with higher GAD-7 scores. Students with no cGPA or a cGPA lower than 3.00 displayed higher GAD-7 scores (13.4 ± 6.2 for no cGPA, 13.0 ± 6.6 for < 3.00 cGPA, P < 0.01). Students clinically diagnosed with a mental disorder reported higher GAD-7 scores (13.6 ± 6.3, P < 0.01), as did those suspecting an undiagnosed mental disorder (14.7 ± 5.3, P < 0.01).

**Table 5:** Significant results for factors associated with GAD-7 scores (*N* = 431).

Variable		GAD-7 score	P-value
**Gender**	Male	7.8 ± 6.4	<0.01
	Female	11.7 ± 6.4	
**Ethnicity**	Saudi	12.1 ± 6.6	<0.01
	Non-Saudi	9.4 ± 6.5	
**Current academic year**	1	13.1 ± 6.3	<0.01
	2	9.4 ± 6.4	
	3	10.2 ± 6.8	
	4	8.5 ± 6.5	
	5	8.3 ± 6.4	
	6	7.2 ± 5.2	
**cGPA**	No cGPA	13.4 ± 6.2	<0.01
	< 3.00	13.0 ± 6.6	
	3.00–3.24	10.1 ± 6.7	
	3.25–3.49	9.6 ± 6.4	
	3.50–3.74	9.2 ± 6.6	
	3.75–4.00	8.0 ± 6.3	
**Clinically diagnosed with a mental disorder**	Yes	13.6 ± 6.3	<0.01
	No	9.9 ± 6.6	
**Suspect having an undiagnosed mental disorder**	Yes	14.7 ± 5.3	<0.01
	No	7.6 ± 6.0	

Data reported as mean GAD-7 score ± SD.

**Figure 2:**
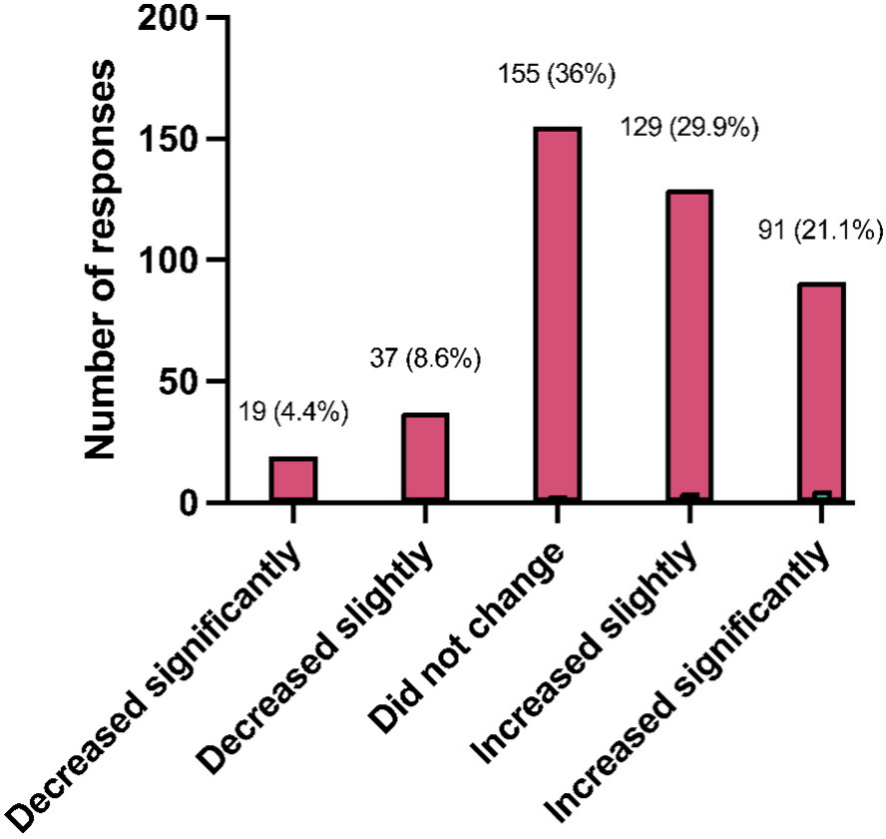
Changes in anxiety levels due to the COVID-19 pandemic quarantine (*N* = 431).

The pandemic’s impact was reflected in the increased anxiety levels during quarantine, reported by 51.0% (220/431) of respondents, while a decrease was noted by 13.0% (56/431) only (**Figure 2**). When asked why it increased, medical students attributed this to a variety of factors. Key reasons include the stress of being confined at home, often in a toxic environment, and the fear of COVID-19 exposure or transmitting the virus to loved ones. The academic stress of constantly studying, especially for board exams, was compounded by the lack of social interaction and outdoor activities. Other factors contributing to heightened anxiety include the monotonous and repetitive nature of staying at home, overthinking due to increased free time, and concerns about the future, including career opportunities and board exams. Additionally, concerns about uncertainty, boredom, and lack of control over their lives were common themes. Despite these challenges, some students found positive aspects in the situation, such as the comfort of studying from home, reduced social and academic pressures, and more time for family and personal growth. The transition to online learning and a change in routine provided a more relaxed environment and opportunities for introspection and pursuing hobbies, which contributed to decreased anxiety for these individuals.

Interestingly, a larger portion of students (53.8%, 232/431) reported not experiencing stress due to the fear of contracting COVID-19. Most students (68.9%, 297/431) indicated difficulties in concentrating on their studies due to the transition to online teaching. More than three-quarters of students (77.0%, 332/431) reported difficulty sleeping due to the pandemic. Responses regarding exam environment preferences were fairly evenly distributed (**Table 6**).

**Table 6:** Effect of COVID-19 pandemic on stress and learning (*N* = 431).

Question/Response	*n* (%)
Q1. Has the idea of getting infected with COVID-19 caused you any stress?	
Yes	199 (46.2)
No	232 (53.8)
Q2. Did you have any difficulty sleeping due to the pandemic?	
Yes	332 (77.0)
No	99 (23.0)
Q3. Did you have any difficulty in concentrating on your studies due to the transition to online teaching?	
Yes	297 (68.9)
No	134 (31.1)
Q4. Putting aside the fear of getting infected with COVID-19, in which environment were you LESS anxious/stressed when writing an exam?	
At university	171 (39.7)
At home	151 (35.0)
No preference	109 (25.3)

**Figure 3:**
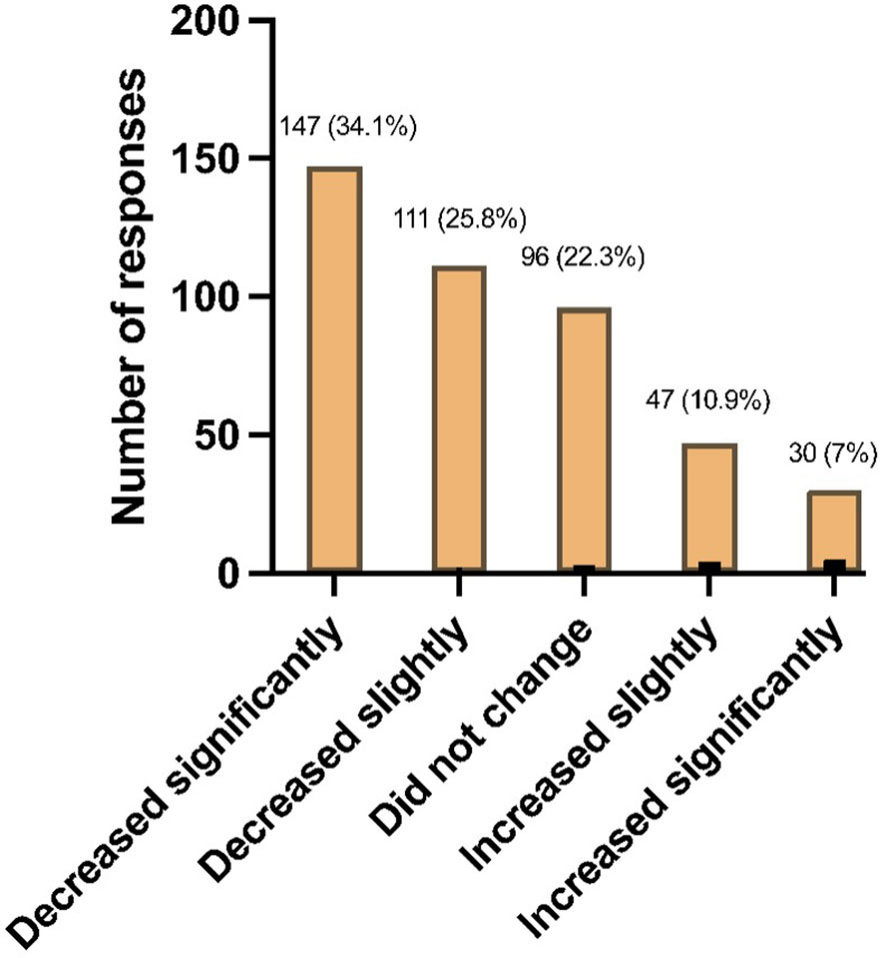
Changes in knowledge retention with the transition to online teaching (*N* = 431).

In **Figure 3**, 59.8% (258/431) of students reported a decrease in information retention due to online learning, with 34.1% (147/431) specifically reporting a significant decrease. Further analysis revealed that first-year students were significantly more negatively affected compared to other years (P < 0.01).

**Figure 4:**
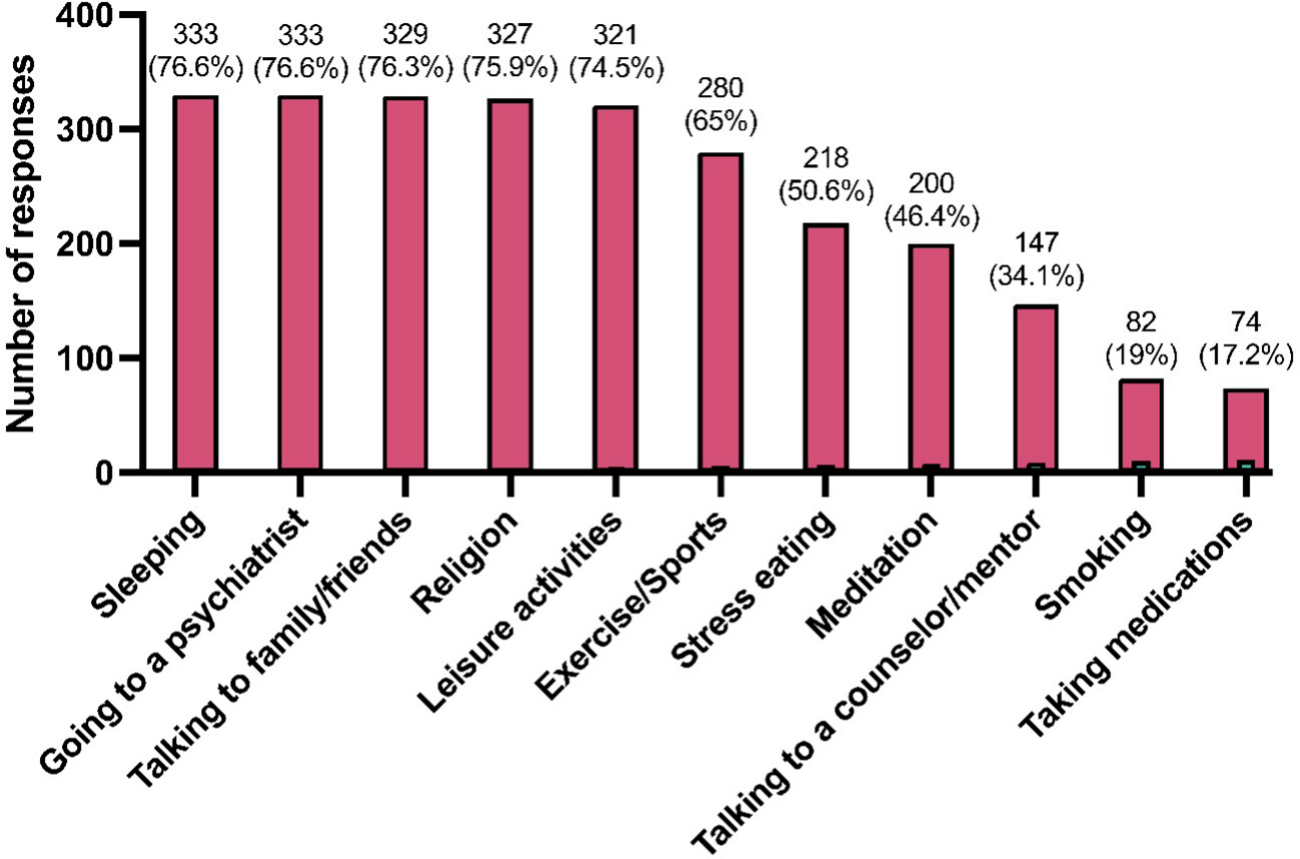
Methods of stress relief reported by the students (*N* = 431).

**Figure 5:**
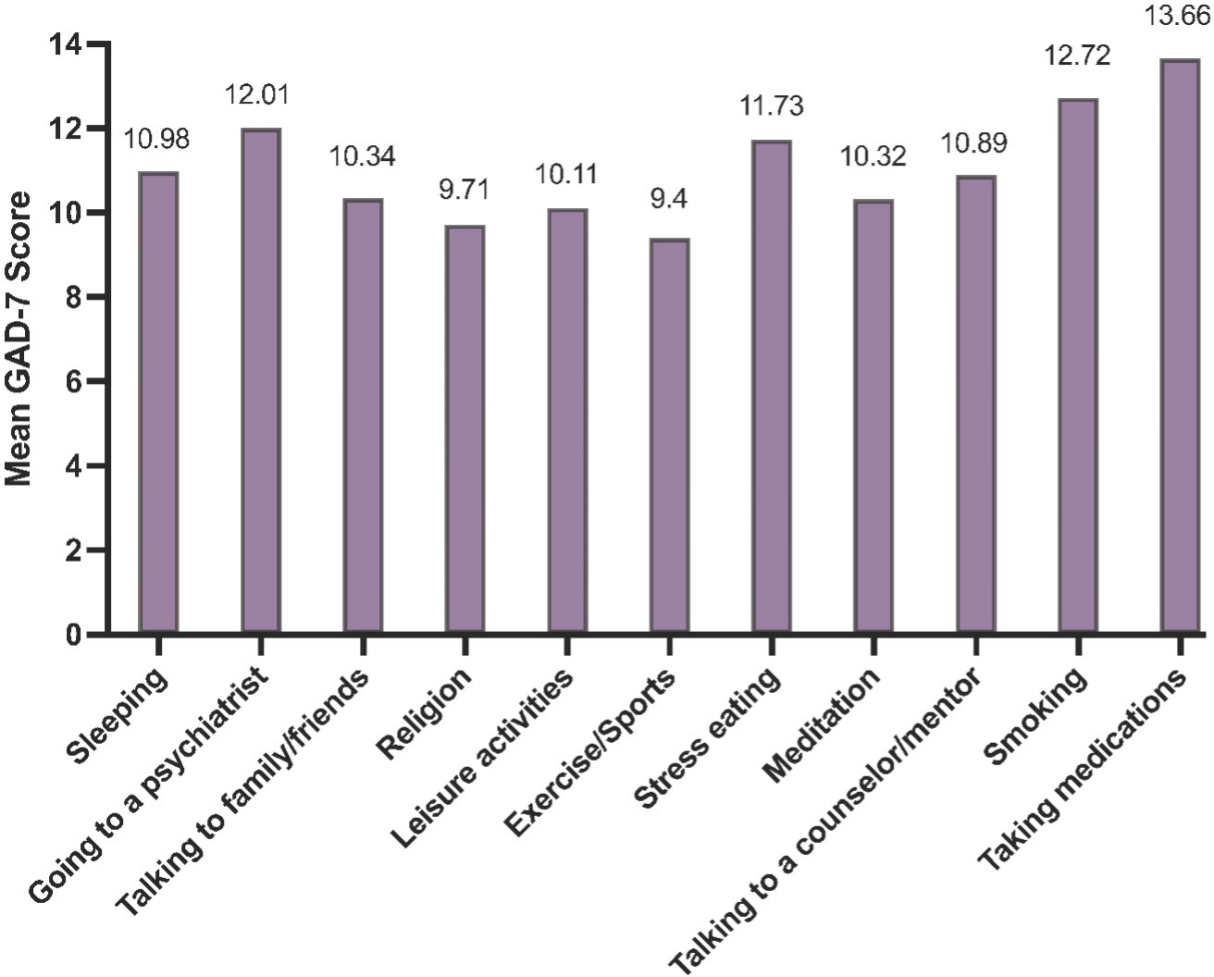
Methods of stress relief according to the average GAD-7 scores (*N* = 431).

**Figure 4** lists various methods of stress relief reported by the respondents. The most used methods are ‘Sleeping’, ‘Going to a psychiatrist’, ‘Talking to family/friends’, and ‘Religion’, each reported by approximately 75% of participants. **Figure 5** correlates the average GAD-7 scores with the methods of stress relief. The highest average GAD-7 scores were associated with ‘Taking medications’ (13.66) and ‘Smoking’ (12.72), while ‘Exercise/Sports’ (9.4) and ‘Religion’ (9.71) are associated with the lowest average GAD-7 scores.

Several key themes emerged from the 233 student responses to our open-ended question regarding reasons that may be responsible for the prevalence of anxiety in medical students studying in Saudi Arabia. A significant factor contributing to anxiety is the academic and financial pressure, particularly due to the high cost and competitive nature of private medical colleges, leading to concerns about family financial burdens, delayed graduation, and fears of academic failure. Students also cited high expectations set by universities, with a rigorous and dense curriculum that offers little time for relaxation, leading to an exam-centric learning environment that lacks enjoyment. The curriculum’s misalignment with crucial future examinations like the United States Medical Licensing Examination, continuous assessments, and irrelevant elective courses further exacerbate stress levels. The COVID-19 pandemic has notably impacted students’ anxiety, primarily due to reduced clinical exposure and hands-on experience, preparing them inadequately for future professional challenges. Social and personal factors, such as family pressure, fear of failure, comparison with peers, and the challenges of living alone, especially for non-local students, significantly contribute to the students’ anxiety. Online learning, adopted due to the pandemic, has led to decreased information retention and concentration, with freshmen facing particular difficulties due to limited peer interaction. The responses also pointed to a pervasive stigma around mental health in the country, making it challenging for students to seek help. A lack of institutional support, including insufficient mental health counseling, lack of recreational activities, and the absence of necessary breaks in the academic schedule, was a recurring theme. Finally, the intense academic competition, stress about future career prospects, and general pressures of medical education compound the anxiety experienced by these students.

Finally, 215 students provided their input regarding what can be changed to improve mental wellbeing. The most suggested method was providing on-campus psychologists and psychiatrists for in-house student support. Key suggestions include academic adjustments such as offering second chances for improved grades, reducing the number and clustering of exams, and revising the GPA system towards a more lenient Pass/Fail approach. Enhanced support and counseling are crucial, with an increased role for mentors, more accessible counseling services, and faculty training in mental health awareness. Students advocate for curricular reforms that lessen exam difficulty and introduce leisure and relaxation events, alongside reevaluating fee structures and expanding scholarships. The establishment of wellness-focused infrastructure, like gyms and recreational spaces, is seen as vital for stress alleviation. Systemic changes are also recommended, including policy reforms for more student-friendly exam schedules and a participatory approach in academic decision-making. Additionally, there’s a call for psychological support through increased mental health awareness and professional psychiatric assistance, coupled with organizing activities for relaxation and fostering a positive, flexible academic environment.

## 4 Discussion

While this study initially aimed simply to assess the prevalence of mental health conditions in medical students, the results have unveiled intriguing patterns that contribute to our understanding of the relationship between mental health conditions and various demographic and academic factors. The overall impact of the pandemic on students’ mental health, encompassing a mix of academic, social, and personal challenges. highlight its complex and multifaceted nature. The recommendations brought up by the students highlight the necessity for universities to adopt a holistic approach in addressing student mental health, balancing academic rigor with supportive and student-centric policies and resources.

In alignment with the data from the Saudi National Mental Health Survey on Saudi youth (24), our results indicate a higher prevalence of GAD and mean GAD-7 scores among Saudi students compared to non-Saudis. Additionally, Saudi students were more likely to be diagnosed with or suspect undiagnosed mental disorders, which resonates with the broader context of mental health in Saudi Arabia (25). The idea that Saudis have a higher-than-average prevalence of mental health conditions is supported by multiple studies that reveal an abnormally high rate of mental health conditions in the Saudi population. For instance, one study found a 28.5% prevalence of mental disorders in primary healthcare patients (26), and another reported a 48% prevalence in a suburban population (27). In fact, a meta-analysis on global prevalence of anxiety among medical students showed that Middle Eastern countries had higher prevalence estimates compared to others (3). The Saudi National Mental Health Survey also highlighted that nearly two in five Saudi youth meet criteria for a mental health condition at some point in their lives, with only a small fraction (13.6%) seeking treatment annually (28).

Conversely, Alyami et al. (2021) found higher rates of depression and anxiety among non-Saudi working-class individuals, suggesting that socioeconomic and living conditions, particularly during the COVID-19 pandemic, might influence mental health (29). These contradicting results may be explained by the difference in the study sample, as Alyani et al.’s study had far more participants from the working class compared to university students, and a large amount of employed non-Saudis are working in the labor force and may live in heavily crowded areas. These living conditions during the COVID-19 pandemic were associated with higher rates of infections, which may explain the high prevalence of anxiety and depression reported in this study (30).

The association between mental health conditions and academic performance was also noteworthy. Students with cGPAs below 3.00 were more likely to have diagnosed or suspect having undiagnosed mental disorder, consistent with Bruffaerts et al. (2013), who observed a 0.2–0.3 drop in cGPA among students with mental health conditions (31). Additionally, first-year students, who typically experience heightened stress and pressure and do not have a cGPA yet, showed higher GAD-7 scores and prevalence of suspected undiagnosed conditions, aligning with findings that college freshmen are more susceptible to mental health issues (32–34). This can potentially be attributed to transitional stress, as students go directly from high school to medical school, and adjustment to a new academic and social environment (35). **Table 6** reveals several associations with GAD-7 scores, including higher scores in female students, aligning with existing literature that suggests females are generally more prone to anxiety disorders than males (36, 37).

A significant association between living alone and the presence of mental health conditions was also observed, resonating with studies on older adults that link solitary living to increased depressive (33) and anxiety (38) symptoms. This suggests a potential correlation even in younger populations (39).

Regarding the pandemic’s impact (**Table 6** and **Figure 2**), most students reported increased anxiety levels, yet this did not directly translate to anxiety over COVID-19 infection. The fact that more students did not report experiencing stress related to COVID-19 infection fears is a notable finding, as it may suggest effective coping mechanisms or a degree of desensitization to the ongoing pandemic situation. This contrasts with the commonly observed trend of heightened fear and stress due to the pandemic in broader populations (40). This implies that the pandemic’s effects on daily life and rapid adjustments might have been the primary anxiety sources, particularly loneliness and social distancing (41).

The online learning effects during the pandemic highlighted challenges in information retention and concentration, particularly among freshmen. This subjective worsening may be due to the decrease in peer-to-peer interactions both between colleagues and with seniors, which especially affects freshmen and leaves them feeling more lost than they would be otherwise (42). Online learning’s efficacy is supported in literature, especially in pre-clerkship years (43, 44). In fact, a systematic review found online learning to be superior to face-to-face learning in certain situations (44). Nonetheless, our findings suggest that subjective measures like focus and memory retention might differ from objective outcomes like cGPA. This discrepancy underscores the need for more nuanced research into online learning’s impacts, particularly in adapting to its benefits without losing face-to-face learning’s advantages. Online learning remains a relatively new system that needs rigorous studying to optimize the learning methods and materials and maximize the effectiveness without compromising the benefits associated with face-to-face learning (45).

Interestingly, the methods of stress relief (**Figures 4**, **5**) showed that medication and smoking, though less frequently used, were linked to higher GAD-7 scores. In contrast, exercise, religious activities, and leisure, more commonly reported, were associated with lower scores, underscoring the potential effectiveness of these methods in mitigating stress and anxiety. This observation aligns with the studies by Steptoe et al. (46), who found that exercise and leisure activities are effective in reducing anxiety and improving mental health, and Khantzian et al. (47), who noted the potential risks associated with self-medication practices like smoking for stress relief. These results may be indicative of the effectiveness of these methods for alleviating stress and anxiety and warrant further investigation with studies that target the association between stress relief methods and mental disorders to understand it more extensively.

This study has several limitations that should be considered. As a single-institution study conducted during the COVID-19 pandemic, the generalizability of our findings may be limited. The reliance on self-reported data and the use of the GAD-7 as a screening tool, rather than clinical interviews, may affect the accuracy of our prevalence estimates. Additionally, the cross-sectional design precludes establishing causal relationships between the factors examined and GAD prevalence. Cultural factors and stigma surrounding mental health in Saudi Arabia may have also influenced participants’ responses. Despite these limitations, this study provides valuable preliminary data on GAD prevalence among medical students in Saudi Arabia and highlights areas for future research.

## 5 Conclusion

Our study revealed a higher prevalence of Generalized Anxiety Disorder (GAD) and associated mental health conditions among Saudi medical students, those living alone, and students with lower cumulative GPAs. Notably, female students, freshmen, and Saudis exhibited elevated GAD-7 scores, particularly those with a history of diagnosed mental health conditions. Stress relief methods such as exercise, religious activities, and leisure were linked to lower GAD-7 scores, suggesting their effectiveness in mitigating anxiety. The transition to online learning was perceived to negatively impact students’ focus and information retention. Given these findings, we advocate for expansive research across Saudi Arabia’s medical student population to uncover broader patterns and inform targeted mental health support strategies.

## Data availability statement

The raw data supporting the conclusions of this article will be made available by the authors, without undue reservation.

## Ethics statement

The studies involving humans were approved by Alfaisal University Institutional Review Board. The studies were conducted in accordance with the local legislation and institutional requirements. The participants provided their written informed consent to participate in this study.

## Author contributions

NA: Conceptualization, Data curation, Methodology, Writing – original draft, Writing – review & editing. TA: Formal analysis, Writing – original draft, Writing – review & editing. AD: Data curation, Writing – original draft. BA: Data curation, Writing – original draft. OB: Data curation, Investigation, Writing – review & editing. RT: Data curation, Writing – review & editing. EB: Data curation, Writing – review & editing. MA: Data curation, Investigation, Writing – review & editing. AOb: Data curation, Writing – review & editing. AOu: Data curation, Investigation, Writing – review & editing.

## Supplementary material

Supplementary File 1 The survey circulated to medical students at Alfaisal University.
