# Supplementary material for: Assessing the prevalence of generalized anxiety disorder in a multicultural medical education setting in Saudi Arabia during the COVID-19 pandemic
Source: Front Psychiatry. 2024 Jul 19;15:1359348. doi: 10.3389/fpsyt.2024.1359348 (PMC11294106; doi:10.3389/fpsyt.2024.1359348)
Supplement: Supplementary File 1 — The survey circulated to medical students at Alfaisal University. [file Table_1.docx]

Supplementary Material

# Survey

1. How old are you?
   ________________________
2. What is your gender?
   1. Male
   2. Female
3. How would you describe your ethnic background?
   1. Saudi
   2. Non-Saudi
4. What is your academic year?
   1. Year 1
   2. Year 2
   3. Year 3
   4. Year 4
   5. Year 5
   6. Year 6
5. What is your cGPA?
   1. I have no GPA (Direct & UPP 1st year students)
   2. <2.00
   3. 2.00-2.24
   4. 2.25-2.49
   5. 2.50-274
   6. 2.75-2.99
   7. 3.00-3.24
   8. 3.25-3.49
   9. 3.50-3.74
   10. 3.75-4.00
6. How would you describe your usual living condition during the academic year?
   1. With Family
   2. With Roommate(s)
   3. Alone
7. Have you been clinically diagnosed with any form of mental disorder?
   1. Yes
   2. No
8. If yes, please specify the condition
   ______________________________________________________________________________
9. Do you feel like you are struggling with any form of mental disorder (not diagnosed)?
   1. Yes
   2. No
10. If yes, please specify the condition
    ______________________________________________________________________________
11. How did your anxiety level change due to the quarantine?
    1. Decreased significantly
    2. Decreased slightly
    3. Did not change
    4. Increased slightly
    5. Increased significantly
12. If it changed, why?
    ____________________________________________________________________________________________________________________________________________________________
13. Has the idea of getting infected with COVID-19 caused you any stress?
    1. Yes
    2. No
14. Did you have any difficulty sleeping due to the pandemic?
    1. Yes
    2. No
15. How has your ability to retain information obtained from lectures changed with regard to online teaching?
    1. Decreased significantly
    2. Decreased slightly
    3. Did not change
    4. Increased slightly
    5. Increased significantly
16. Did you have any difficulty in concentrating on your studies due to the transition to online teaching?
    1. Yes
    2. No
17. Putting aside the fear of getting infected with COVID-19, in which environment were you LESS anxious/stressed when writing an exam?
    1. At home
    2. At university/C-DEX
    3. No preference
18. If you felt anxious or were going through a stressful period, which of the following measures
    1. Exercise/Sports
       1. Yes
       2. No
    2. Talking to a counselor/mentor
       1. Yes
       2. No
    3. Taking medications
       1. Yes
       2. No
    4. Smoking
       1. Yes
       2. No
    5. Religion
       1. Yes
       2. No
    6. Talking to family/friends
       1. Yes
       2. No
    7. Stress eating
       1. Yes
       2. No
    8. Sleeping
       1. Yes
       2. No
    9. Going to a psychiatrist
       1. Yes
       2. No
    10. Leisure activities (e.g. video games, painting, reading)
        1. Yes
        2. No
    11. Meditation
        1. Yes
        2. No
19. GAD-7: Over the last 2 weeks, how often have you been bothered by the following problems?
    1. Feeling nervous, anxious, or on edge
       1. 0 (Not At All)
       2. 1 (Several Days)
       3. 2 (Over Half the Days)
       4. 3 (Nearly Every Day)
    2. Not being able to stop or control worrying
       1. 0 (Not At All)
       2. 1 (Several Days)
       3. 2 (Over Half the Days)
       4. 3 (Nearly Every Day)
    3. Worrying too much about different things
       1. 0 (Not At All)
       2. 1 (Several Days)
       3. 2 (Over Half the Days)
       4. 3 (Nearly Every Day)
    4. Trouble relaxing
       1. 0 (Not At All)
       2. 1 (Several Days)
       3. 2 (Over Half the Days)
       4. 3 (Nearly Every Day)
    5. Being so restless that it’s hard to sit still
       1. 0 (Not At All)
       2. 1 (Several Days)
       3. 2 (Over Half the Days)
       4. 3 (Nearly Every Day)
    6. Becoming easily annoyed or irritable
       1. 0 (Not At All)
       2. 1 (Several Days)
       3. 2 (Over Half the Days)
       4. 3 (Nearly Every Day)
    7. Feeling afraid as if something awful might happen
       1. 0 (Not At All)
       2. 1 (Several Days)
       3. 2 (Over Half the Days)
       4. 3 (Nearly Every Day)

(Developed by Drs. Robert L. Spitzer, Janet B.W. Williams, Kurt Kroenke, and colleagues.)

1. If any of the above problems were identified, how difficult have these made it for you to do your work, take care of things at home, or get along with other people?
   1. Not Difficult at all
   2. Somewhat Difficult
   3. Very Difficult
   4. Extremely Difficult
2. From your experience, mention a few reasons that may be responsible for the prevalence of anxiety in medical students studying in Saudi Arabia
   ____________________________________________________________________________________________________________________________________________________________
3. In your opinion, mention a few methods by which the university can assist medical students with their mental wellbeing
   ____________________________________________________________________________________________________________________________________________________________
